# Direction selectivity in the visual system of the zebrafish larva

**DOI:** 10.3389/fncir.2013.00111

**Published:** 2013-06-18

**Authors:** Christoph Gebhardt, Herwig Baier, Filippo Del Bene

**Affiliations:** ^1^Institut Curie, Centre de RechercheParis, France; ^2^CNRS UMR 3215Paris, France; ^3^INSERM U934Paris, France; ^4^Department Genes – Circuits – Behavior, Max Planck Institute of NeurobiologyMartinsried, Germany

**Keywords:** visual system, direction selectivity, zebrafish, optic tectum, neural circuits

## Abstract

Neural circuits in the vertebrate retina extract the direction of object motion from visual scenes and convey this information to sensory brain areas, including the optic tectum. It is unclear how computational layers beyond the retina process directional inputs. Recent developmental and functional studies in the zebrafish larva, using minimally invasive optical imaging techniques, indicate that direction selectivity might be a genetically hardwired property of the zebrafish brain. Axons from specific direction-selective (DS) retinal ganglion cells appear to converge on distinct laminae in the superficial tectal neuropil where they serve as inputs to DS postsynaptic neurons of matching specificity. In addition, inhibitory recurrent circuits in the tectum might strengthen the DS response of tectal output neurons. Here we review these recent findings and discuss some controversies with a particular focus on the zebrafish tectum’s role in extracting directional features from moving visual scenes.

## INTRODUCTION

Extracting motion information from a visual scene is a key ability of most visual systems throughout the animal kingdom. Moving objects change their position over time in reference to the animal and thus project onto the retina as both spatial and temporal patterns of varying light intensities. With regard to motion detection, an important parameter that can be extracted from these patterns is the direction of a moving stimulus. This information is of vital importance for specific behaviors such as prey capture, collision avoidance, or escape from a predator.

Detailed information has been gathered in insects and mammals about motion processing, but studies were mostly restricted to the computations performed by the sensory surface, i.e., small retinal circuits (Elstrott and Feller, 2009; Borst and Euler, 2011; Wei and Feller, 2011). How direction-selective (DS) is attained and processed by higher brain areas is less evident. Studies addressing this question in the mammalian visual cortex have often investigated either single neurons by electrophysiology or columns of many hundred or thousand of cells by optical imaging, thus lacking the resolution necessary to ask circuit-level questions (Bonhoeffer and Grinvald, 1991; Chapman et al., 1996; Priebe et al., 2010).

The optic tectum of larval zebrafish is an excellent brain structure to study motion processing at a higher circuit-level. The tectum is the main retinorecipient brain region and homologous to the superior colliculus in mammals. Sitting at the surface of the brain, it is easily accessible to a wide variety of techniques, including electrophysiology, laser ablations, optogenetics, and optical imaging. In addition to receiving a majority of retinal afferents, the tectum is an integrator of sensory information from multiple modalities (Meek, 1983; Vanegas and Ito, 1983). Main areas of the tectum can be histologically distinguished. The *stratum* *periventriculare* (SPV) contains the cell bodies of most tectal neurons (periventricular neurons, PVNs) whereas the synaptic neuropil area contains the PVNs’ dendrites and axons as well as the axons of retinal ganglion cells (RGCs). The tectal neuropil is a precisely laminated structure within which the RGC axons mostly target the superficial layers (Xiao et al., 2005): the *stratum opticum* (SO), right beneath the basement membrane, and the *stratum fibrosum et griseum superficiale* (SFGS). Classical Golgi studies in adult goldfish and genetic single-cell labeling in larval zebrafish revealed that the PVNs have a single dendritic shaft that extends into the tectal neuropil, often crossing multiple layers (Vanegas et al., 1974; Meek and Schellart, 1978; Scott and Baier, 2009; Nevin et al., 2010; Robles et al., 2011).

Importantly, zebrafish are also genetically accessible rendering them well suited for functional studies of the visual system that require targeting of protein-based indicators to genetically identified subpopulations of neurons. This opens up the exciting possibility of studying DS processing across specific neuronal populations, often with single-cell resolution.

## DEVELOPMENT OF DS IN THE OPTIC TECTUM APPEARS TO BE GENETICALLY HARDWIRED

The anatomical and morphological development of the zebrafish larval visual system has been investigated in great detail (e.g., Stuermer, 1988). Between 34 and 48 hours post fertilization (hpf) retinal axons leave the retina and start invading the tectal neuropil. By 72 hpf, retinal axons have sparsely innervated the entire tectum and begin to form terminations at their topographically correct targets. At around the same time, the lens has developed to produce a focused image within the photoreceptor layer of the retina (Easter and Nicola, 1996). After tectal coverage is achieved, dendritic arbors undergo remodeling until a relatively stable state is reached around 7 days post fertilization (dpf). The laminar development of retinotectal wiring seems to be largely independent of externally evoked visual activity. Activity-dependent mechanisms, however, influence the refinement of the RGC arbors that form the visuotopic map (Stuermer et al., 1990; Gnuegge et al., 2001; Hua et al., 2005; Smear et al., 2007; Nevin et al., 2008; Fredj et al., 2010).

Extraction of directional information from a visual scene requires that DS neurons exhibit an asymmetric response to visual stimuli that move in the preferred vs. the opposite (null) direction. This functional asymmetry must ultimately be a consequence of an asymmetry in wiring, regulation of synaptic strengths, or dendritic conductance. How does this asymmetry come about during development? Several possibilities have been proposed. For one, it could be that this asymmetry of DS circuits is genetically hardwired, for instance by cell-surface molecular cues that act upon dendrite or synapse distribution and are expressed very early in visual system development. It is also possible that DS circuits show initially non-asymmetric responses and are subsequently biased in one direction by activity-dependent mechanisms. Of course, genetic hardwiring and activity-based mechanisms might also act in concert to shape the final DS response of neurons of the visual system.

In a landmark study, Niell and Smith (2005) used *in vivo *Ca^2^^+^ imaging with the synthetic Ca^2^^+^ indicator OGB1-AM for an initial functional description of the entire tectal cell population during development. Among other visual parameters, the authors also examined DS in the tectum. They reported that a large proportion of tectal cells were already DS as early as 72 hpf and DS reached nearly mature levels after 78 hpf. This is perhaps surprising considering that during that time window the very first retinal axons have barely reached their termination zones in the tectum and retinotectal circuits are still undergoing thorough refinement. Furthermore, zebrafish larvae that were reared completely in the dark showed normal DS responses, which were indistinguishable from larvae reared under default light–dark cycle conditions.

The latter finding is not consistent with a study in *Xenopus* tadpoles (Engert et al., 2002). This paper reported that DS of tectal cells was not apparent at early developmental stages but extensive training with a moving stimulus was able to induce DS responses in a few recorded tectal neurons, suggesting an experience-dependent mode of DS development. This discrepancy between zebrafish and *Xenopus* could be due to a true species difference as others (Podgorski et al., 2012) have also found DS plasticity after visual training in tadpoles. However, it might also be possible that in tadpoles, DS of tectal cells is present at early stages and repeated training generated short-lasting single neuron and/or network connectivity changes that obscured the initially hardwired tuning of the recorded tectal cells.

Niell and Smith’s findings were, however, largely confirmed and extended, by a later study (Ramdya and Engert, 2008). Normally, retinal projections to the tectum are completely crossed, i.e., tectal neurons are monocular. By surgically removing a single tectal lobe the authors partially re-routed the retinal projection to the ipsilateral tectum, thereby generating a few binocularly innervated tectal cells (i.e., neurons that responded to inputs from both eyes). They found that these binocular cells showed the same DS response to moving stimuli when these were presented to either eye. Furthermore, depriving the animals from any externally evoked visual activity by rearing them in the dark, showed again no difference in the development of the observed binocular DS compared to light-reared larvae. This is in agreement with the experience-independent DS development observed earlier by Niell and Smith (2005). Furthermore, since it is also unlikely that correlated intrinsic activities between the two eyes occur, this might indicate that the development of direction selectivity in zebrafish is, in addition to being experience-independent, also independent of spontaneously generated retinal waves (Wong et al., 1993).

Taken together the available evidence suggests that, at least in zebrafish, direction selectivity is established at the earliest stage measurable and develops independently of activity in the visual system.

## TECTUM-INTRINSIC COMPUTATION OF DIRECTION SELECTIVITY

**FIGURE 1:**
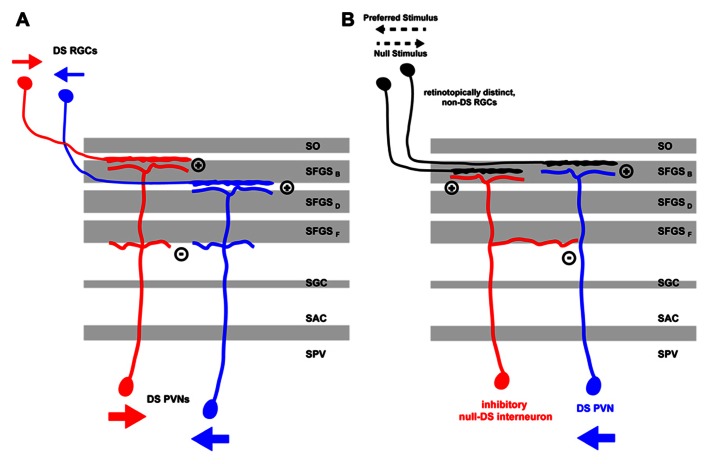
**Mechanisms for direction selectivity computation in the zebrafish larva tectum.** **(A)** Direction information from a moving stimulus is extracted by retinal circuits and transferred by RGC axons, which are specific for the stimulus direction (small red or blue arrows), to distinct laminae in the tectal neuropil. The retinal arbors are then targeted by the distal arbors of PVNs in their respective laminae thus acquiring direction specificity themselves. Heterotypic connections between proximal PVN arbors might lead to reciprocal inhibition thus sharpening DS PVN response to a moving stimulus in a specific direction (large red or blue arrows). **(B)** A DS PVN (blue) receives excitatory inputs from one or more non-DS RGCs in the tectal neuropil. In addition, it receives intratectal inhibitory input from an interneuron (red) that is retinotopically positioned on the side of the DS PVN facing the preferred stimulus direction. Thus, a moving stimulus in the preferred direction (black pointed arrow) elicits excitatory currents in the retina that excite DS PVNs. Currents from the inhibitory interneurons arrive later and do not interfere with the PVNs activity state. Moving stimuli in the null-direction, however, elicit inhibitory currents in the tectal interneuron, which arrive first at the DS PVN thus blocking any subsequent excitatory currents the DS PVN might receive from the retina. DS, direction-selective; RGC, retinal ganglion cell; PVN, periventricular neuron; SAC, stratum album centrale; SFGS (B-F), sublaminae of stratum fibrosum et griseum superficiale; SGC, stratum griseum centrale; SO, stratum opticum; SPV, stratum periventriculare.

Functional models of DS differ mainly in how excitatory or inhibitory input currents are tuned (i.e., to the preferred vs. the null-direction) and then integrated in time to finally give rise to tectal DS outputs. In the zebrafish, there is evidence for two different DS mechanisms being implemented. For one, DS could be predominantly computed by retinal circuits, which then would drive postsynaptic DS neurons directly via direction-tuned excitatory inputs. Alternatively, excitatory retinal input might show rather weak DS and subsequent tectal recurrent inhibition, tuned to the null-direction, might shape the final PVN response in the preferred direction of the stimulus. Both of these mechanisms are not mutually exclusive and might be implemented in a complementary fashion in retina and tectum (**Figure 1**).

In the above-mentioned study, Ramdya and Engert (2008) found evidence for a null-inhibition mechanism in the zebrafish tectum. By using the surgically induced binocular retinotectal circuit they addressed if direction selectivity is computed in the retina and then projected into pre-specified tectal modules or if, alternatively, tectal inhibitory circuits perform this computation. Two lines of evidence suggested that the latter mechanism is at work. First, the authors performed an experiment in which they displayed a visual stimulus consisting of two stationary spots separated in time and jumping between different visual field positions of one eye. These two stationary spots are seen as apparent motion and elicit a DS response in a subset of the recorded tectal neurons. Showing these two spots with a slight delay to the left and right eye of fish with binocular input to the tectum, should not elicit any DS response if direction selectivity relies purely on retinal computation. However, the authors observed that some binocular tectal cells were showing a response to an apparent-motion stimulus that was comparable to the stimulus applied to the contralateral eye alone. Second, a pharmacological block of tectal inhibition by injection of bicuculline in the tectum led to a loss of DS in most of the tectal neurons under normal conditions. This was due to the response to the null-direction being strongly increased after drug injection.

Taken together, these results provided evidence for a tectal DS computing mechanism involving strong recurrent inhibition tuned to the null-direction rather than direct retinal excitatory currents. It is unclear, however, if the artificially altered circuit is indeed indicative of how direction selectivity is computed under normal, unaltered conditions or if the apparent-motion stimulus is at all suited for investigating feature extraction from a moving visual scene. For instance, if the retinal inputs to the recorded tectal cells are already DS, then activation by the apparent-motion stimulus, even though it may not be the optimal stimulus, will elicit a DS response in the postsynaptic cell. This response can look deceptively similar to a tectum-intrinsic, *de novo* DS computation.

In a follow-up paper, Grama and Engert (2012) analyzed the contribution of excitatory and inhibitory currents to tectal DS in more detail. By patching a random set of tectal neurons in the SPV, they found that excitatory input currents, supposedly originating from the retina, were correlated but not tuned to the direction of the stimulus motion, as measured by counting the spikes evoked by a moving bar. However, inhibitory currents, presumably coming from tectal inhibitory interneurons, were inversely correlated with the direction of motion, i.e., they were biased for the null-direction. Furthermore, the authors observed latencies in the millisecond range between excitatory and inhibitory currents. Inhibitory currents preceded the excitatory ones in the null-direction (median = 39 ms) and vice versa in the preferred direction (median = 157 ms). Based on this evidence, the authors suggested a model in which tectal DS responses are computed from non-DS retinal inputs by tectal recurrent inhibition. For such a mechanism to work, they postulated the existence of a special type of tectal interneuron, which, similar to the starburst amacrine cell in the retina (Fried et al., 2002), is responding to moving stimuli in the null-direction and is asymmetrically connected to DS tectal output neurons (**Figure 1**). This interneuron type should be positioned on the side of the DS cell that represents the preferred direction along the corresponding axis of the retinotopic map. In this configuration, stimuli coming from the null-direction will selectively suppress the response of the output cell. While plausible, there is currently no evidence for the existence of such an asymmetrically organized circuit in the zebrafish tectum.

## TECTAL PROCESSING OF DIRECTION-SELECTIVE RETINAL INPUTS

Differing from an exclusively tectum-intrinsic mechanism for direction computation, two recent studies showed that RGC inputs are already tuned to stimulus direction when they reach the tectum. In the first study, Meyer and colleagues expressed the genetically encoded Ca^2^^+^indicator SypGCaMP3, driven by the *isl2b* promoter, in almost all retinal synapses terminating in the retinorecipient layers of the tectum (Nikolaou et al., 2012). By statistical analysis of activity distribution maps from stacks of several animals over several experimental trials, they found three major RGC DS input clusters in the tectal neuropil, one caudal-to-rostral and two different rostral-to-caudal directed clusters with a down-up or up-down DS component respectively. These DS clusters match the response tuning profiles of the previously reported DS-RGCs in goldfish (Maximov et al., 2005). In the tectum, these inputs were segregated superficially in two discrete layers of the SFGS. Caudal-to-rostral-tuned inputs were distributed more superficially in the tectal neuropil than inputs of the other directions. Notably, these layers are preferentially innervated by RGCs that have bistratified dendrites in the ON and OFF sublayers of the inner plexiform layer of the retina (Robles et al., 2013). RGCs of this class have been shown to be DS in several other vertebrates, including birds, mammals, and fish.

Furthermore, Nikolaou et al. (2012) reported that retinal synaptic inputs responding to caudal-to-rostral motion predominated quantitatively over those responding to rostral-to-caudal motion, which is consistent with previous studies (Maximov et al., 2005; Niell and Smith, 2005). Moreover, the authors found two clusters of orientation-selective (OS) presynaptic inputs (horizontal and vertical motion in both directions) that spanned several laminae in the middle layers of the neuropil and were well separated from DS inputs in the superficial neuropil. Surprisingly, they observed also a retinotopic bias of the observed DS and OS clusters. The DS inputs were mostly confined to the posterior half of the tectum while the two OS clusters were distributed anteriorly and posteriorly, respectively. It is currently unclear if these distributions reflect the existence of topographically restricted RGC populations, retinotopic differences in the retinal circuits or presynaptic modulation within the tectum. Considering that these cumulative imaging data were highly processed and thresholded, it is also possible that synapse density of DS cells and thus SypGCaMP3 expression accounted for the observed topographic differences.

The most comprehensive study of DS in the zebrafish retinotectal system so far was recently presented by Bollmann and colleagues (Gabriel et al., 2012). From an enhancer-trap screen, they identified two Gal4-VP16 transgenic lines that labeled subsets of DS interneurons in the tectum. *Tg(Oh:G3) *drives expression of UAS (upstream activation sequence)-linked reporter genes mostly in rostral-to-caudal-tuned cells, whereas *Tg(Oh:G4)* labels caudal-to-rostral-tuned cells. In addition to differences in DS, these two subsets of tectal neurons also differ morphologically. While both are bistratified, they have their distal dendritic arbor in different layers of the tectal neuropil.

Similar to Nikolaou et al. (2012), Gabriel et al. (2012) reported three main types of presynaptic DS inputs (one caudal-to-rostral cluster and two rostral-to-caudal ones) and observed that each targets one specific lamina in the tectal neuropil. In a series of elegant experiments, Gabriel et al. (2012) showed that functionally identified postsynaptic neurons had their dendritic arbors specifically in the very same laminae in the neuropil as the matching DS RGC inputs, lending further weight to their mapping of DS retinal inputs.

While the two studies by Nikolaou et al. (2012) and Gabriel et al. (2012) converge on the same broad conclusions they differ in important details, which appear irreconcilable at first glance. Gabriel et al. (2012) observed one rostral-to-caudal-tuned cluster (with both up-down and down-up DS component) that was situated more superficially in the tectal neuropil than the caudal-to-rostral-tuned cluster. This is the inverse of what Nikolaou and colleagues reported. How can this apparent discrepancy be explained? The precise layout of the laminar distribution of retinal inputs in the tectal neuropil might offer a solution. In a parallel set of studies, utilizing brain bow labeling of RGC axons, Robles et al. (2013) reported that the zebrafish retinotectal neuropil is composed of at least ten laminae. The SO is subdivided into two layers, SO1 and SO2, while the SFGS contains six distinct layers, SFGS1 through SFGS6. Each of the retinotectal layers harbors a complete retinotopic map and is innervated by a distinct combination of RGC types (Robles et al., 2013). In this new scheme, Gabriel et al.’s DS inputs might, for instance, be localized to SO2, SFGS1 and maybe SFGS2, while Nikolaou et al.’s might be in SFGS1 and SFGS2. The two studies would then unequivocally agree that one of the ten layers, most likely SFGS1, is sensitive to caudal-to-rostral direction.

The reason for the differences in both studies could be that weak rostral-to-caudal oriented signals (i.e. rostral-to-caudal signals found in SO2 and SFGS2, respectively) might have been difficult to record: In order to isolate the presynaptic activity in the tectum, Gabriel et al. (2012) used pan-neuronal GCaMP3-expression and subsequent pharmacological blockage of glutamatergic transmission in tectal cells. This approach could have lead to a high intensity background impeding the detection of weak clusters. By contrast, the genetic targeting and/or expression levels of SypGCaMP3 in the study by Nikolaou et al. (2012) might not have been sufficient to reveal all existing retinal laminae. Furthermore, the threshold for identifying DS input signals were set differently in the two studies. This choice might also have contributed to the observed differences.

In summary, a scheme that assumes the existence of three presynaptic layers in the superficial third of the neuropil with alternating DS, a caudal-to-rostral lamina sandwiched between two rostral-to-caudal oriented ones (possibly each of the latter containing two distinct sub-clusters with a down-up or up-down DS component, respectively), might explain the available data.

Furthermore, Gabriel et al. (2012) reported that excitatory inputs, likely from RGCs, determine the DSof at least some tectal cell types. This is in agreement with Nikolaou et al. (2012), but appears to contradict the conclusions of Grama and Engert (2012) who did not find DS-tuned excitatory inputs but emphasized rather the importance of inversely DS-tuned inhibitory intra-tectal currents. Gabriel et al. (2012) also report that the two different types of bistratified DS tectal neurons are GABAergic, inhibitory interneurons. Thus, they suggest that a feed-forward, null-direction inhibition via the proximal dendritic arbors of the cells might serve as a means to fine-tune the tectum’s output. It is still possible that some types of tectal cells are mainly driven by DS excitatory input, whereas others are controlled by DS-tuned inhibitory inputs.

## CONCLUSION AND DIRECTIONS FOR FUTURE RESEARCH

DS neurons are found in several regions along the visual pathway, including retina, tectum, and cortex. It is important to understand how neurons acquire DS characteristics at each of these stages. Studies in zebrafish have revealed that DS is hardwired and can develop independently of patterns of activity. In the tectum, DS retinal inputs terminate in the tectal neuropil in specific laminae, where they form connections with the lamina-restricted dendrites of tectal interneurons. This feed-forward mode of DS wiring is reminiscent of the so-called “labeled lines” that are found in other sensory systems (Kauer and White, 2001). Evidence for the contribution of tectal recurrent connections, especially inhibitory ones, is less clear. If it exists, it might contribute to sharpening the response of DS output neurons.

In conclusion, it seems to us that, for a complete understanding of DS computation, additional genetic markers for functionally identified types of DS neurons are needed, not only in the tectum but also in the retina (Huberman et al., 2009; Gabriel et al., 2012). It will be productive to trace the connections of the different types of DS-RGCs from the retina to the tectal layers and identify their postsynaptic partners. Future research should also elucidate how DS computation is used in behavioral contexts, i.e., how DS information is transferred to motor centers and used to generate oriented behavior toward prey or away from predators. The zebrafish tectum, as a prominent center for sensorimotor transformation in an optically and genetically accessible organism, will be an excellent place to investigate these fundamental questions of systems neuroscience.

## Conflict of Interest Statement

The authors declare that the research was conducted in the absence of any commercial or financial relationships that could be construed as a potential conflict of interest.

## References

[B1] Bonhoeffer T. Grinvald A. (1991). Iso-orientation domains in cat visual cortex are arranged in pinwheel-like patterns. *Nature* 353 429–431 10.1038/353429a0 1896085

[B2] Borst A. Euler T. (2011). Seeing things in motion: models, circuits, and mechanisms. *Neuron* 71 974–994 10.1016/j.neuron.2011.08.031 21943597

[B3] Chapman B. Stryker M. P. Bonhoeffer T. (1996). Development of orientation preference maps in ferret primary visual cortex. *J. Neurosci.* 16 6443–6453 8815923 10.1523/JNEUROSCI.16-20-06443.1996 PMC2669086

[B4] Easter J. Nicola G. N. (1996). The development of vision in the zebrafish (*Danio rerio*). *Dev. Biol.* 180 646–663 10.1006/dbio.1996.0335 8954734

[B5] Elstrott J. Feller M. B. (2009). Vision and the establishment of direction-selectivity: a tale of two circuits. *Curr. Opin. Neurobiol.* 19 293–297 10.1016/j.conb.2009.03.004 19386483 PMC2805110

[B6] Engert F. Tao H. W. Zhang L. I. Poo M. (2002). Moving visual stimuli rapidly induce direction sensitivity of developing tectal neurons. *Nature* 419 470–475 10.1038/nature00988 12368854

[B7] Fredj N. B. Hammond S. Otsuna H. Chien C.-B. Burrone J. Meyer M. P. (2010). Synaptic activity and activity-dependent competition regulates axon arbor maturation, growth arrest, and territory in the retinotectal projection. *J. Neurosci.* 30 10939–10951 10.1523/JNEUROSCI.1556-10.2010 20702722 PMC6634700

[B8] Fried S. I. Münch T. A. Werblin F. S. (2002). Mechanisms and circuitry underlying directional selectivity in the retina. *Nature* 420 411–414 10.1038/nature01179 12459782

[B9] Gabriel J. P. Trivedi C. A. Maurer C. M. Ryu S. Bollmann J. H. (2012). Layer-specific targeting of direction-selective neurons in the zebrafish optic tectum. *Neuron* 76 1147–1160 10.1016/j.neuron.2012.12.003 23259950

[B10] Gnuegge L. Schmid S Neuhauss S. C. F. (2001). Analysis of the activity-deprived zebrafish mutant macho reveals an essential requirement of neuronal activity for the development of a fine-grained visuotopic map. *J. Neurosci.* 21 3542–3548 11331383 10.1523/JNEUROSCI.21-10-03542.2001 PMC6762499

[B11] Grama A. Engert F. (2012). Direction selectivity in the larval zebrafish tectum is mediated by asymmetric inhibition. *Front. Neural Circuits* 6:59 10.3389/fncir.2012.00059 PMC3432856 22969706

[B12] Hua J. Y. Smear M. C. Baier H. Smith S. J. (2005). Regulation of axon growth in vivo by activity-based competition. *Nature* 434 1022–1026 10.1038/nature03409 15846347

[B13] Huberman A. D. Wei W. Elstrott J. Stafford B. K. Feller M. B. Barres B. A. (2009). Genetic identification of an On-Off direction- selective retinal ganglion cell subtype reveals a layer-specific subcortical map of posterior motion. *Neuron* 62 327–334 10.1016/j.neuron.2009.04.014 19447089 PMC3140054

[B14] Kauer J. S. White J. (2001). Imaging and coding in the olfactory system. *Ann. Rev. Neurosci.* 24 963–979 10.1146/annurev.neuro.24.1.963 11520924

[B15] Maximov V. Maximova E. Maximov P. (2005). Direction selectivity in the goldfish tectum revisited. *Ann. N. Y. Acad. Sci.* 1048 198–205 10.1196/annals.1342.018 16154933

[B16] Meek J. (1983). Functional anatomy of the tectum mesencephali of the goldfish. An explorative analysis of the functional implications of the laminar structural organization of the tectum. *Brain Res.* 287 247–297 6362772 10.1016/0165-0173(83)90008-5

[B17] Meek J. Schellart N. A. (1978). A Golgi study of goldfish optic tectum. *J. Comp. Neurol.* 182 89–122 10.1002/cne.901820107 81216

[B18] Nevin L. M. Robles E. Baier H. Scott E. K. (2010). Focusing on optic tectum circuitry through the lens of genetics. *BMC Biol. *8:126. 10.1186/1741-7007-8-126 PMC2949621 20920150

[B19] Nevin L. M. Taylor M. R. Baier H. (2008). Hardwiring of fine synaptic layers in the zebrafish visual pathway. *Neural Dev.* 3 36 10.1186/1749-8104-3-36 PMC2647910 19087349

[B20] Niell C. M. Smith S. J. (2005). Functional imaging reveals rapid development of visual response properties in the zebrafish tectum. *Neuron* 45 941–951 10.1016/j.neuron.2005.01.047 15797554

[B21] Nikolaou N. Lowe A. S. Walker A. S. Abbas F. Hunter P. R. Thompson I. D. (2012). Parametric functional maps of visual inputs to the tectum. *Neuron* 76 317–324 10.1016/j.neuron.2012.08.040 23083735 PMC4516722

[B22] Podgorski K. Dunfield D. Haas K. (2012). Functional clustering drives encoding improvement in a developing brain network during awake visual learning. *PLoS Biol. * 10:e1001236 10.1371/journal.pbio.1001236 PMC3254648 22253571

[B23] Priebe N. J. Lampl I. Ferster D. (2010). Mechanisms of direction selectivity in cat primary visual cortex as revealed by visual adaptation. *J. Neurophysiol.* 104 2615–2623 10.1152/jn.00241.2010 20739595 PMC2997030

[B24] Ramdya P. Engert F. (2008). Emergence of binocular functional properties in a monocular neural circuit. *Nat. Neurosci.* 11 1083–1090 10.1038/nn.2166 19160507 PMC2958220

[B25] Robles E. Filosa A. Baier H. (2013). Precise lamination of retinal axons generates multiple parallel input pathways in the tectum. *J. Neurosci.* 33 5027–5039 10.1523/JNEUROSCI.4990-12.2013 23486973 PMC3641828

[B26] Robles E. Smith S. J. Baier H. (2011). Characterization of genetically targeted neuron types in the zebrafish optic tectum. *Front. Neural Circuits * 5:1 10.3389/fncir.2011.00001 PMC3046383 21390291

[B27] Scott E. K. Baier H. (2009). The cellular architecture of the larval zebrafish tectum, as revealed by Gal4 enhancer trap lines. *Front. Neural Circuits * 3:13 10.3389/neuro.04.013.2009 PMC2763897 19862330

[B28] Smear M. C. Tao H. W. Staub W. Orger M. B. Gosse N. J. Liu Y. (2007). Vesicular glutamate transport at a central synapse limits the acuity of visual perception in zebrafish. *Neuron* 53 65–77 10.1016/j.neuron.2006.12.013 17196531 PMC1828615

[B29] Stuermer C. A. (1988). Retinotopic organization of the developing retinotectal projection in the zebrafish embryo. *J. Neurosci.* 8 4513–4530 2848935 10.1523/JNEUROSCI.08-12-04513.1988 PMC6569580

[B30] Stuermer C. A. Rohrer B Münz H. (1990). Development of the retinotectal projection in zebrafish embryos under TTX-induced neural-impulse blockade. *J. Neurosci.* 10 3615–3626 2230950 10.1523/JNEUROSCI.10-11-03615.1990 PMC6570109

[B31] Vanegas H. Ito H. (1983). Morphological aspects of the teleostean visual system: a review. *Brain Res.* 287 117–137 10.1016/0165-0173(83)90036-X 6315186

[B32] Vanegas H. Laufer M. Amat J. (1974). The optic tectum of a perciform teleost I. General configuration and cytoarchitecture. *J. Comp. Neurol.* 154 43–60 10.1002/cne.901540104 4815183

[B33] Wei W. Feller M. B. (2011). Organization and development of direction-selective circuits in the retina. *Trends Neurosci.* 34 638–645 10.1016/j.tins.2011.08.002 21872944 PMC4538999

[B34] Wong R. O. L. Meister M. Shatz C. J. (1993). Transient period of correlated bursting activity during development of the mammalian retina. *Neuron* 11 923–938 10.1016/0896-6273(93)90122-8 8240814

[B35] Xiao T. Roeser T. Staub W. Baier H. (2005). A GFP-based genetic screen reveals mutations that disrupt the architecture of the zebrafish retinotectal projection. *Development* 132 2955–2967 10.1242/dev.01861 15930106

